# Novel loss-of-function variants of *TRAPPC2* manifesting X-linked spondyloepiphyseal dysplasia tarda: report of two cases

**DOI:** 10.1186/s12881-019-0802-2

**Published:** 2019-05-03

**Authors:** Joon Yeon Won, Dayeon Kim, Seon Young Park, Hye Ran Lee, Jong-Seok Lim, Jong Hoon Park, Mi Hyun Song, Hae Ryong Song, Ok-Hwa Kim, Yonghwan Kim, Tae-Joon Cho

**Affiliations:** 1 Department of Biological Sciences, Sookmyung Women’s University, 100 Cheongpa-ro 47-gil, Yongsan-gu, Seoul, 04310 Republic of Korea; 2 Division of Pediatric Orthopaedics, Seoul National University Children’s Hospital, 101 Daehak-ro, Jongno-gu, Seoul, 03080 Republic of Korea; 3 Department of Orthopaedic Surgery, Korea University Guro Hospital, 148 Gurodong-ro, Guro-gu, Seoul, 08308 Republic of Korea; 4 Department of Radiology, Woorisoa Children’s Hospital, 15 Saemal-ro, Guro-gu, Seoul, 08291 Republic of Korea; 5 Department of Orthopaedic Surgery, Seoul National University College of Medicine, 101 Daehak-ro Jongno-gu, Seoul, 03080 Republic of Korea

**Keywords:** X-linked spondyloepiphyseal dysplasia tarda, TRAPPC2, Skeletal dysplasia, Gene expression

## Abstract

**Background:**

X-linked spondyloepiphyseal dysplasia tarda (SEDT-XL) is a skeletal disorder characterized by defective structures of vertebral bodies and/or of epiphyses of the long bones, resulting in moderately short stature and early joint degeneration. *TRAPPC2* gene, which is important for collagen secretion, has been reported as causative for SEDT-XL.

**Case presentation:**

Here, we report two variants of *TRAPPC2* gene of SEDT-XL patients, a missense variant of start codon, c.1A > T, and a deletion variant, c.40delG. To understand molecular consequence of the variants, we establish an in vitro gene expression assay system and demonstrate that both mutated genes are transcribed, but are not properly translated, indicative of the pathogenic nature of those *TRAPPC2* variants.

**Conclusions:**

In the current study, we provide additional experimental data showing that loss-of-function *TRAPPC2* variants are probably causative for SEDT-XL phenotype. These findings further contribute to the understanding the clinical picture related to *TRAPPC2* gene.

## Background

X-linked spondyloepiphyseal dysplasia tarda (SEDT-XL) is a skeletal dysplasia affecting male subjects, characterized by defective structures of vertebral bodies and/or of epiphyses of the long bones, leading to short stature and premature joint degeneration [1–3]. SEDT-XL has been linked with variants in transport protein particle complex subunit 2 (TRAPPC2) [1, 4, 5]. TRAPPC2 protein consists of 140 amino acids without described enzymatic domains. To date, 32 insertion or deletion, 10 splicing, 9 nonsense, and 6 missense variants of *TRAPPC2* gene in SEDT-XL patients have been listed in the Human Gene Mutation Database (http://www.hgmd.cf.ac.uk/ac/index.php) [6]. Based on the reported pathogenic variants in *TRAPPC2* in different ethnic groups, it was suggested that there is no specific population with increased risks for inherited SEDT-XL [7]. Most variants are predicted to cause premature truncation. However, only a few of the variants were studied for its functional defect of protein such as c. 93 + 5G > A or p.D47Y [2, 3, 8, 9]. Interestingly, locations of *TRAPPC2* variants are not necessarily related to the phenotype of affected SEDT-XL individuals. In one study, a phenotype of a patient with a deletion of 19 amino acids was not significantly distinguishable from that of another patient with a deletion of 71 amino acids [3, 4]. Although it still remains elusive how the phenotype of SEDT-XL patients are linked to loss of TRAPPC2 functions, it was proposed that loss of TRAPPC2 might affect either the Golgi integrity [3] or the collagen secretion level [2]. We previously reported a missense variant at the start codon (c.1A > T) [10], and detected a novel single nucleotide deletion (c.40delG) in patients presented with SEDT-XL phenotype. In this study, we investigated and found that both variants are transcribed, but they are not expressed at all, as confirmed by immunoblotting, implying that loss of TRAPPC2 function is causative for SEDT-XL phenotypes of these patients.

## Case presentation

**Fig. 1:**
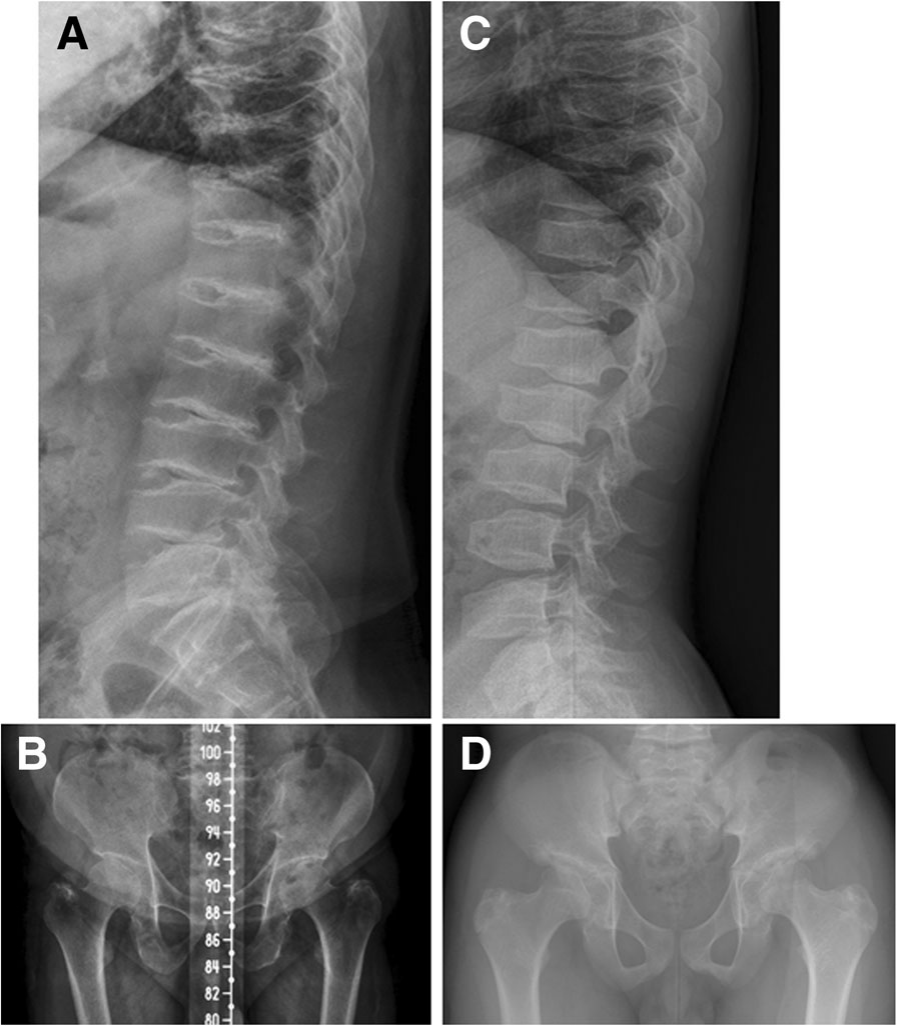
Lateral spine and hip radiographs of two SEDT-XL patients. **a**, **b** Radiographs taken from the proband with the c.1A > T, *TRAPPC2* variant at age of 45 years. **a** Thoracolumbar lateral spine shows uniform platyspondyly and hump-like bony projections at the centroposterior portions of upper and lower endplates, and narrowing or almost obliteration of the intervertebral disc spaces. **b** Pelvis radiograph shows narrow iliac wings with relatively long ischium and pubic bones, deep acetabulum and degenerative osteoarthtis with narrow hip joints. **c**, **d** Radiographs from the proband with the c.40delG, *TRAPPC2* variant at age 15 years. **c** Spine lateral radiograph shows platyspondyly with elongated vertebral bodies and hump-like bony protrusion at the upper and lower endplates, dominantly noted in the lumbar spine. **d** Hip shows relatively long ischium and pubic bones. Deep acetabulum and dysplastic femoral heads with premature degenerative hip joint narrowing are noted

Approval to perform this study was obtained from the institutional review board of Seoul National University Hospital, Seoul, Republic of Korea. Two cases of Korean SEDT-XL patients with East Asian ethinicity, were found to harbor sequence variation of *TRAPPC2* gene. The first patient, a 49-year-old man was referred for right hip pain starting from mid-thirties with abnormal radiographic findings. His height was 138 cm (z-score − 6.2) and arm span 152 cm. Short neck and increased anteroposterior diameter of the thoracic cage were noted. The height and body proportion of the parents and three siblings of the proband were in normal range. Radiographs revealed generalized platyspondyly with hump-like protruding at the endplates of the lumbar spine on the lateral view, a diagnostic finding of SEDT-XL (Fig. 1a-b). Hip radiograph showed degenerative osteoarthritis of the hip joint resulting in profound hip joint space narrowing. Target sequencing including *TRAPPC2* and subsequent Sanger sequencing revealed a hemizygous sequence variation of c.1A > T, predicting p.Met1Lys. His mother could not be examined for mutation as she was not alive. The second patient was a 15-year-old boy referred for short stature and back pain. His clinical finding was previously reported [11]. His height was 146.8 cm (z-score − 4.3). Lateral spine radiographs showed humps at the posterior half of the endplates, similar to the first patient and informative finding for the diagnosis of of SEDT-XL(Fig. 1c-d). Sanger sequencing for *TRAPPC2* revealed a hemizygous sequence variation, c.40delG, predicting p.Asp14Ile*fs*X27, which was inherited from the mother. She was short (146 cm, z-score − 3.12), but did not show any radiographic abnormality, back pain or joint pain [11]. Both variants were not annotated in dbSNP, 1000 Genomes database, the Genome Aggregation Database (gnomAD), NHLBI Exome Sequencing Project Exome Variant Server (EVS), or Exome Aggregation Consortium (ExAC) Browser.

**Fig. 2:**
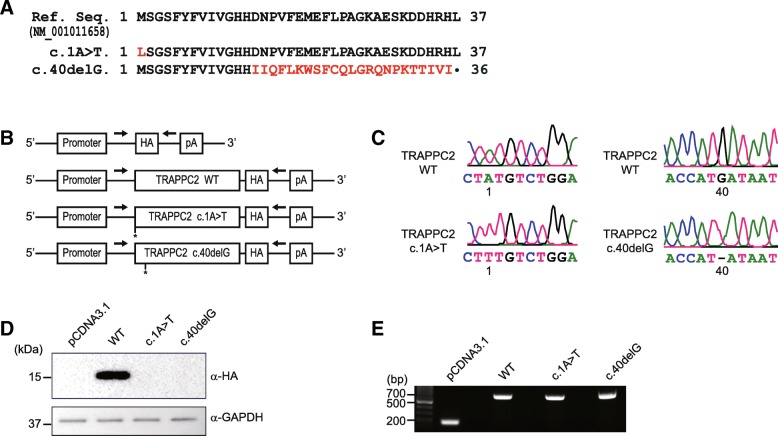
Functional analysis of *TRAPPC2* variants found in the SEDT-XL patients (**a**) Predicted amino acid sequences of the *TRAPPC2* variants found in the SEDT-XL individuals. The c.1A > T missense variant would change the starting codon Methionine to Leucine. The deletion variant, c.40delG, would only produce the first 13 amino acids correctly and then abruptly terminate the translation. The gene accession number for human *TRAPPC2* mRNA is indicated. **b** DNA constructs used in this study. Wild-type (WT) or the *TRAPPC2* variants are expressed under the CMV promoter. HA tag is introduced at the C-terminus of the cDNAs and thus the HA tag does not affect translation of the *TRAPPC2* variants. The arrows indicate primers for RT-PCR. These primers are specific to the vector used in this experiment to exclude any endogenous *TRAPPC2* genes. **c** Chromatogram displaying the mutations in the TRAPPC2 expression vectors. A missense mutation at the position 1 showed a change of base from adenine to thymine in the left pair. A deletion of guanine at the position 40 is demonstrated in the right pair. **d**-**e** Analyses of the *TRAPPC2* variant expression at protein and transcript level. 293 T HEK cells were transfected with individual pCDNA3.1, WT, or TRAPPC2 variant expressing vector, and cell lysates were subjected to Western blot analysis with HA antibody (**d**) and used to prepare total RNA for RT-PCR (**e**). The size markers for DNA fragments and proteins are indicated

The first variant, c.1A > T, misses its translational start codon, and consequently, it would not be translated. However, it is possible to use the in-frame cryptic initiating ATG codon, located 57 nucleotides downstream from the start codon, which might lead to express the N-terminal truncated TRAPPC2 mutant if translated (Fig. 2a). The predicted protein from the second mutation, c.40delG, is C-terminal truncated mutant, p.Asp14Ile*fs*X27 (Fig. 2a). In an attempt to understand molecular consequences of the identified *TRAPPC2* variants, we established the in vitro experimental system, which enabled us to determine expression of both TRAPPC2 variants at the level of transcription and translation. To this end, we cloned wild-type (WT) *TRAPPC2* cDNA from the cDNA library prepared from U2OS cells. Each mutation was introduced using QuikChange II XL Site-Directed Mutagenesis Kit (Agilent). To study expression levels of mutants, WT and two *TRAPPC2* variants were constructed to the pcDNA3.1 plasmid of which the HA tag was added to 3′ end to each cDNA to prevent translation of HA from causing expression of mutants that would otherwise be not translated (Fig. 2b). After obtaining each plasmid, we confirmed that the variants were correctly reproduced in the vector by Sanger sequencing (Fig. 2c). Next, to determine expression levels, 293 T cells were transfected with control, wild-type or mutant TRAPPC2 expression vector individually, and 24 h later, the prepared cell lysates were fractionated with immunoblot analysis to characterize expression patterns of those two mutants. As shown in Fig. 2d, only WT was detected; the missense mutation, c.1A > T, and the frameshift mutation, c.40delG, failed to express the TRAPPC2 mutant. No proteins expression could be due to either no translation or no transcription. To test if mutants were transcribed or not, we performed reverse transcription polymerase chain reaction (RT-PCR). We designed a set of primers to detect TRAPPC2 transcripts produced by the vectors only and to exclude the endogenous TRAPPC2 transcripts (arrows in the Fig. 2b). Cell lysates for RT-PCR were prepared in the same way as for immunoblotting. After harvesting and lysing cells, lysates were first used to isolate total mRNA with RNeasy Mini Kit (Qiagen) according to a given instruction. Then, the purified RNA was subjected to produce cDNA library with reverse transcriptase reaction (SuperScript III First-Strand Synthesis Kit, Thermo). Next, using the cDNA library, we performed PCR reaction with primers that specifically cover the TRAPPC2 cDNA produced by the transformed vectors. When the PCR products were run on an agarose gel, both WT and two mutants revealed a band at size of 700 bp (Fig. 2e), suggesting two possible scenarios: the first is that two *TRAPPC2* variants are transcribed, but not translated. The other is that these variants are transcribed and translated, but the translated proteins are degraded.

## Discussion and conclusions

We anticipated that both mutant TRAPPC2 proteins would be expressed at a smaller size if they were translated at all. In c.1A > T mutant, the initial starting codon is missing, but there is another in-frame ATG codon 57 nucleotides downstream of the start codon; it may lead to the N-terminal truncated TRAPPC2 transcript at a smaller size. Another variant, c.40delG, causes premature termination at amino acid 37, and thus if expressed, it would produce a protein that is about a third of WT in size. However, our data demonstrated that both mutants were transcribed, but the translation products of these mRNA are not properly translated as it either lost the start codon or is likely degraded by nonsense-mediated RNA decay. Absence of any protein would likely cause significant changes in cellular processes, which would be followed by developmental problems or disorder. In the current study, we provide experimental evidences showing that two *TRAPPC2* variants identified from SEDT-XL patients lead to no TRAPPC2 protein production. Although here we showed that loss-of-function TRAPPC2 variants is causative for the SEDT-XL, further studies will be required to understand molecular mechanisms of pathophysiology of the disease.

Both SEDT-XL individuals showed defects in skeletal development and have been through difficulties due to the abnormalities as described above. Complete absence of a single protein, especially when it is involved in a crucial pathway, should take a toll on a cell, and on an individual, possibly lethally. However, our data shows that two SEDT-XL individuals are survived without the TRAPPC2 protein, although they both had abnormal bone structures and related pain, suggesting existence of an alternate protein or a pathway that takes over a role of TRAPPC2. TRAPPC2 is associated with numerous proteins, either directly or indirectly [12]. If it is not expressed, and yet affected individuals can be survived, then there is highly likely another protein that compensates for the absence of this multi-interacting protein, and identifying the substituting protein would be helpful in gene therapies of SEDT-XL. One possible set of candidates is pseudogenes of *TRAPPC2*. There are nine pseudogenes, and the most likely compensator is *TRAPPC2B* (also known as *SEDLP1*); it is located on chromosome 19q13.4 and has potential to produce a protein identical to that coded by *TRAPPC2* [13]. Since *TRAPPC2B* is not on X chromosome, and yet can encode an identical protein, it is highly possible that individuals with *TRAPPC2* variants who survived absence of the protein may compensate by utilizing *TRAPPC2B*. Individuals with *TRAPPC2* variants can restore the pathway of protein transport by a means of *TRAPPC2B* if they inherited both normal copies of chromosome 19. However, if either one or both copies of *TRAPPC2B* are damaged, then, the normal pathway would be only partially overcome, or not at all, resulting in different degrees of phenotypes in patients.

## Abbreviations

cDNA: complimentary deoxyribonucleic acid
HA: Hemagglutinin
mRNA: messenger ribonucleic acid
RT-PCR: Reverse transcription polymerase chain reaction
SEDT-XL: X-linked spondyloepiphyseal dysplasia tarda
*TRAPPC2*: Transport protein particle complex subunit 2
WT: Wild-type
